# Homeopathy—A lively relic of the prescientific era

**DOI:** 10.1007/s00508-023-02164-w

**Published:** 2023-03-24

**Authors:** Yannick Borkens, Udo Endruscheit, Christian W. Lübbers

**Affiliations:** 1 INH—Informationsnetzwerk Homöopathie, GWUP e. V., Arheilger Weg 11, 64380 Roßdorf, Hessen Germany; 2 Charité, Charitéplatz 1, 10117 Berlin, Berlin Germany; 3 Humboldt-Universität zu Berlin, Unter den Linden 6, 10117 Berlin, Berlin Germany; 4 HNO Weilheim, Pöltnerstr. 22, 82362 Weilheim, Bayern Germany

**Keywords:** Alternative medicine, Brief Report, Evidence-based medicine, Homeopathy, Scientific integrity

## Abstract

Homeopathy was first postulated by the German physician Samuel Hahnemann in 1796 and 220 years later homeopathy is the most popular and widespread alternative medicine. Partly, it is also part of the national healthcare and insurance systems but homeopathy is not without controversy within the medical and healthcare community. Its implausible basic assumptions, some of which contradict natural laws, do not lead us to expect that its remedies have any specific effect. In fact, there is no study or systematic review to date that reliably certifies homeopathy to have an effect beyond the placebo effect and other context effects. In this respect it must be disconcerting how widely homeopathy is applied and represented in therapeutic practice. It indeed claims a role within scientific (evidence-based) medicine but cannot substantiate this claim. It displays clear characteristics of pseudoscience [1]. This implies a lot of problems, such as misleading people and tackling medical ethics up to scientific publication practices. Furthermore, it turns out that quite a few people do not know exactly what homeopathy is, which may lead them to make wrong decisions for their personal health. This article summarizes the information about homeopathy and its problematic implications and serves as a general introduction to this topic and its unacceptable role in today’s medicine.

The medical irrelevance of the sham method of homeopathy has been proven with more than sufficient probability [2]. As a major testimonial, the statement “Homeopathic products and practices” of the European (EASAC 2017) can be regarded. The primary aim of this brief report is therefore not to take another look at homeopathy from a medical scientific perspective, but rather focus attention on the implications of the still continuous and largely uncritically accepted existence of this method in medical practice, in the medical scientific sphere and in the judgement of the general public.

## Homeopathy and the basic assumptions

Homeopathy is still the most popular alternative healing method, even partly integrated into healthcare systems and in many cases also considered and applied as a therapy option by academically trained doctors. Considering the prescientific origin of the method and its long-proven irrelevance in medical science, this is a phenomenon that should not be regarded indifferently. Many people trust homeopathy for various ailments, minor as well as more serious diseases, but what is homeopathy? It often turns out that most people who swear by homeopathy have little or no knowledge of what it is about at all.

### What is homeopathy?

Homeopathy is an alternative healing method of German origin. The method was postulated by the German physician and pharmacist Samuel Hahnemann (1755–1843) at the end of the eighteenth century. In Hufeland’s *Journal of Practical Medicines *[3] Hahnemann 1796 presented his leading idea, the principle of similarity (*Similia similibus curentur*) for the first time. He invented this principle as the central point of his construct in the first edition of his Organon in 1810.

However, the basic assumptions on which homeopathy rests are either refuted or implausible. The method is based on two basic principles, the similarity principle and the dilution principle.

- The similarity principle, *similia similibus curentur* (similar things may be healed by similar things) states that a substance that causes symptoms (the symptom bundle) in healthy people can be used as a medicine against just these symptoms in people suffering from them. This is a special expression of the ancient anthropocentric doctrine of signatures or similarities, which on an esoteric basis assumed references between natural phenomena and human concerns. In Hahnemann’s teaching, this comes to life for the last time in a powerful way in the transitional period from the prescientific to the scientific era. Hahnemann did not focus on a typifying concept of disease as used by modern etiology medicine as the basis of diagnosis and therapy. Rather, he made the individual symptom picture of the individual patient the goal of his homeopathic therapy. He believed that *nothing more could be recognised of the disease than the outwardly visible symptoms*. Consequently, during his lifetime he never named a disease. How wrong this is can be seen from the fact that symptoms can be appropriate reactions of the organism to disease as well as expressions of organ and functional disorders as a result of progressive disease. Homeopathy is not able to differentiate here and does not want to.
- The dilution principle states that the efficacy of similar substances, which according to the similarity principle is regarded as healing for the respective ailments, is increasingly enhanced the higher it is diluted (to the point of impossible detectability). Homeopathy states that it is “more than mere dilution” because the process is called potentization. Through ritual shaking (succussion) during the gradual dilution process, hitherto “dormant hidden medicinal powers” are, in Hahnemann’s words, supposed to gradually and increasingly pass from the substances into the solvent. Hahnemann imagined a gradual “disappearance” of the hitherto exclusively material substance. Indeed, the substance disappears in the remains of the respective dilution processes. The esoteric content of this postulate, which contradicts natural law, is obvious. Nevertheless, a very popular and failing explanation of this procedure is the so-called water memory [4].

Today, homeopathy is one of the most popular therapeutic methods worldwide. An estimated 500,000 physicians worldwide have additional homeopathic training, 45,000 of them in Europe alone [5]. Despite these benefits and its popularity with patients, homeopathy is more than controversial. This is mainly as to date there has been no proof that homeopathy is effective [6]. Yet the criticism of homeopathy is not new. Homeopathy was criticised from the beginning in the slowly dawning era of scientific questioning. In 1825, the physician Johann Christian Heinroth published his work *Anti-Organon oder das Irrige der Hahnemannischen Lehre im Organon der Heilkunst* (*Anti-organon or the erroneous nature of the Hahnemanian doctrine in the organon of the art of healing*), in which he anticipated many of today’s arguments of science-based criticism of homeopathy. In America, as early as 1853, the physician P. Dyer wrote in the Boston Medical and Surgical Journal (today known as The New England Journal of Medicine) [7]:

Among the various systems of medicine now prevalent in the community, none seems to be so utterly absurd or so supremely ridiculous as that termed “homoeopathy”.

Unlike many other methods from the prescientific era, homeopathy still holds its own today. The reasons for this are manifold and largely sociocultural rather than medical. In view of its medical scientific invalidity, however, it should not do so under the label of medicine and especially not with the seal of public health systems. Because of this, the scientifically oriented critique of homeopathy has succeeded more and more in recent years in attracting public attention to the problem of homeopathy [8]. These demands are also increasingly reaching the nonscientific mainstream, especially via the media [9].

### Who discovered homeopathy?

Samuel Hahnemann’s (1755–1843) invention of homeopathy was intended to serve as a counter-concept to classical medicine at that time. Medicine was then based on the so-called humoral pathology, which attributed diseases to an imbalance of the four humours, following the ancient physician Galenus (born 129 AC, date of death unknown). Hahnemann saw these therapies, with some justification, as a method that did more harm than good and often weakened patients to the point that they died from the therapy rather than from their disease. He referred to these methods as allopathy, a deliberately delimiting and pejorative term that has incomprehensibly survived to this day as an occasional name for scientific medicine. Important discoveries of modern medicine, such as the discovery of cellular pathology or the discovery of disease-causing microbes (pathogens) took place in part centuries after Hahnemann’s death in 1843 [10].

Hahnemann was born in Meissen, Saxony and studied medicine in Leipzig. During his studies, he also made translations of medical and physiological works into German. In 1777 he became a Freemason. Also in 1777, he accompanied the Freiherr Samuel von Brukenthal to Hermannstadt (Transylvania, Romania) as a tutor and personal physician. He stayed there for 2 years and apparently observed numerous cases of malaria. In 1779 he completed his medical studies with a doctorate at the Friedrich-Alexander-Universität in Erlangen, Bavaria. His concept of homeopathy probably dates back to his time in Hermannstadt. Here he had the experience that was to lead him to his thoughts on homeopathy. He took cinchona bark in a self-experiment and describes the symptoms as follows: *Die Füße, die Fingerspitzen, u. s. w. wurden mir erst kalt, ich ward matt und schläfrig, dann fing mir das Herz an zu klopfen, mein Puls ward hart und geschwind, eine unleidliche Ängstlichkeit, ein Zittern (aber ohne Schauder), eine Abgeschlagenheit durch alle Glieder; dann ein Klopfen im Kopfe, Röthe der Wangen, Durst, kurz alle mir sonst beim Wechselfieber gewöhnlichen Symptome erschienen nacheinander; doch ohne eigentlichen Fieberschauder* (My feet, fingertips, etc., first became cold, I became dull and sleepy, then my heart began to beat, my pulse became hard and rapid, an unpleasant anxiety, a trembling (but without shivering), a lassitude through all the limbs; then a throbbing in the head, redness of the cheeks, thirst, in short, all the symptoms otherwise usual to me in changeable fever appeared one after the other but without an actual shivering fever) [4]. This description is considered today as the first step in the development of homeopathy. According to this teaching, cinchona bark will be a possible medicine against malaria. He subsequently transferred his apparent experience of the simile from the cinchona experiment to other substances. When used on the sick, he attributed healing powers to them against the symptoms that these substances were supposed to trigger in healthy people. He believed that he found a divine law through this healing with the like. The entire homeopathic teaching is based on this law.

To this day, it has not been possible to reproduce this experiment of Hahnemann. In Hahnemann’s time, the cause of malaria was still unknown. It was not until 1880 that *Plasmodium* was discovered as the causative agent of malaria by Alphonse Laveran. In 1897, Ronald Ross found that *Plasmodium* was transmitted by mosquitoes of the genus *Anopheles*. Both received the Nobel Prize in Medicine or Physiology for their work (Ross in 1902, Laveran in 1907). Homeopathy on the other hand is still without a Nobel Prize today.

A general principle of similarity is still unknown to science today and has not been necessary for any explanation of a natural phenomenon so far. As already mentioned above, it is a reminiscence of old, partly esoteric ideas of the prescientific era and cannot claim any validity, which is fatal for homeopathy, because it loses its cornerstone.

## Why is homeopathy criticism important?

First of all, the terms myth and evidence should be explained: myth is derived from the Greek *mythos* (μúθος = sound, speech, tale, legendary story) and in its original meaning is a narrative. These often make a claim of validity for the truth they assert. For the ancient sophists (pre-Socratic philosophers), myth stands in contrast to *logos* (λόγος = reason, language, speech, proof, tenet, doctrine, sense, reason), which attempts to substantiate its claims through evidence. Evidence, which derives from the Latin *evidentia*, in common denotes that which is unquestionable to the eye and is thus opposed to myth. In modern medicine, as well as in modern philosophy of science, in a stricter definition evidence refers to empirical findings that confirm scientific theories or refute their attempts at confirmation. On this basis, evidence-based medicine claims that patient-centered decisions should be made in medical treatment based on the best available empirical evidence of the effectiveness of the procedures used [8]. This contrasts with alternative procedures such as homeopathy, for which efficacy has not been proven to date. Its principles do not stand up to comparison with well-established scientific knowledge, nor can they empirically demonstrate a specific benefit, which is shown by a series of systematic reviews including a plethora of clinical studies (the body of evidence) [11].

Given the popularity of homeopathy as probably the best-known and most widespread method of alternative medicine, people often ask why criticism of this method is important or why it is voiced at all. After all, critics could simply ignore homeopathy; however, the situation is much more complex. There are many arguments in favor of criticizing homeopathy. In the following, four points should be brought closer to the reader. These are only examples and do not completely represent all important arguments in the homeopathy debate [12].

### Homeopathy and its impact on the public

- Therapy procrastination: a relevant criticism of homeopathy is the so-called therapy procrastination. Although homeopathic remedies are usually used for minor illnesses such as colds or minor injuries, it also happens that homeopathic remedies are used for more serious illnesses, such as cancer or severe infections such as coronavirus disease 2019 (COVID-19). This is precisely one of the effects of the very popularity and good reputation of homeopathy, its perception as a serious and proven healing method in the general public and medical professionals alike. This use leads to the aforementioned protraction of therapy. This means that meaningful and effective therapies are delayed and made more difficult (possibly unsuccessful) if therapy attempts are first made with homeopathy. Particularly with severe illnesses with a small therapeutic time window, such as cancer, a therapy begun too late may have severe effects.
- Medication affinity: less medically, but more educationally relevant is the so-called medication affinity. As already described above globules are often used to treat minor injuries such as abrasions (arnica globules). One can imagine that guardians, for example, quickly reach for these when their child is injured on a playground. Such minor pediatric aches and pains that certainly do not require the administration of a drug. This may cause a lifelong medication affinity because children in particular can be quickly and permanently conditioned to such behavior.
- Health insurance: in Germany, some health insurance companies cover (partly) the cost of homeopathics. Yet other services such as dentures or visual aids, i.e., services that have empirically proven effects, are not (fully) covered. In this way, not only is money wasted but homeopathy is also attributed an effect that the insured persons perceive as credible, but that does not exist in this way.
- Conspiracy theories: this is following the takeover of homeopathy by health insurance companies and the resulting credibility effect. This fuels conspiracy theories and fake science regarding homeopathy. For example, the narrative of the gentle alternative to pharmaceutical products of the negatively connoted alleged *BigPharma* is very deliberately cultivated (David and Goliath effect). This conceals the fact that homeopathics such as globules and others are also produced and distributed by these, partly large, profit-oriented industrial companies. The relevance of such distorted information is shown by political efforts in Europe to treat homeopathy as a possibly serious alternative solution in the context of antibiotic-resistant germs. These tendencies and based-on decisions in turn bring more misinformation about homeopathy into the race and keep the merry go round of constantly new finally meaningless studies on homeopathy alive, which then in turn make the general public believe in a non-existent scientific character of the method. A vicious circle that must be named as such and stopped [13]. This requires clear positions from science, politics and also the media that leave no doubt about the medical irrelevance of homeopathy.

### Homeopathy and its impact on scientific integrity

Despite the complete implausibility of its basic assumptions and the negative results of empirical studies and systematic reviews, homeopathy claims a role in medicine and especially in medical science. The fact that it continues to be given credit in many ways, and that it enjoys a public reputation not least for this reason, is an untenable state of affairs from the point of view of rationally based science. Criticism of homeopathy can therefore not be limited to informing patients/consumers but must also clearly show how much pseudo-medicine has infiltrated the medical sphere in the case of homeopathy and that this can no longer be justified. This brief report is intended to contribute to this.

Several scientific journals practice in particular repeatedly panders to homeopathy by publishing studies on homeopathy, preferably with positive results, despite its claim to scientific integrity. One may ask how this is possible (as long as it does not concern journals that are to be assigned to the homeopathic sphere from the outset).

On the one hand, it may be that articles on homeopathy are peer-reviewed by homeopaths because journals wrongly assume that they are the “experts” for homeopathic studies. That this will only exponentially increase the confirmation bias that is apparently overlooked. The tendency to want to publish sensational results as a priority may also play a role.

Furthermore, the obvious disinterest of the professional community in publications on homeopathy is an understandable but very regrettable circumstance. Of course, there is a broad consensus in the scientific world that homeopathy is medically irrelevant; however, scientists and researchers understandably rarely take the time and trouble to counter this to the necessary extent, especially in public.

Last but not least, it is also a problem that the research results of homeopathy rarely if ever have any influence on the therapeutic practice of this bogus method. Homeopathy does not “need” empirical studies. Everything that is necessary has been established by Hahnemann: the suitability of substances as homeopathic remedies is determined by the principle of similarity and the chosen remedy by the individual symptom picture of the patient. The correct application in individual cases is a matter for the “true healing artist” who is convinced of the method. The “studies”, be they clinical investigations or so-called basic research, which above all seek to prove specifics in highly diluted solutions, serve far more the self-assurance of homeopathic circles, the impressing of the inclined public and the urge to advance to scientific reputation.

One may ask oneself what this has to do with medical science. It is therefore not only a contribution to active patient protection and health literacy but also in the own interest of medical science and practice not to tolerate such tendencies any longer and to give them a clear public rejection.

### Homeopathy in the media

Medical topics have always been transported by the media and made accessible to the public. Over time, similar to scientific medicine, a contrast developed between popular, pseudo and purely scientific positions and publications. The implementation of modern German drug legislation in 1978 endowed homeopathy with a special role that not only allowed easy (market) accessibility (as a drug without proof of efficacy) but also gave it massive public credibility that was unique among alternative healing methods [14]. Until the 2010s, the German medical profession still spoke of a pluralism of medicine. The goal of this pluralism was to put the former understanding of a mere empirical medicine (based mainly on the experience of the unique therapist) on an equal level with evidence-based modern medicine (based on clinical trials). Logically, these efforts also influenced the reporting media landscape [15].

Today, more realistic points of view have emerged in the media landscapes. Orientation to scientific evidence concepts has become the standard for adequate medical reporting for quite some time. This may have been overlooked by the mainstream of homeopathy-friendly reporting in the past. One can occasionally get the impression from the media coverage that it is about a feuilletonistic rather than a medical topic, all the more so as it is scientifically closed [15].

A major factor in the change in the media landscape regarding homeopathy is the public discourse that has intensified in recent years, leading to much stronger criticism and its public perception. Coverage of homeopathy has changed noticeably since then, and the criticisms of science-based homeopathy sceptics began to find entrance. Nevertheless, we are still far from a consistent evidence-based account of homeopathy as a whole. A cause that should not be underestimated is also the still powerful influence of the homeopathic advocacy group, which still succeeds in constructing and presenting a mimicry of science, not to say denialism [13]. According to this, the appearance is maintained that there is still an unresolved scientific dissent to the evidence base. The stakes of politics should also not be underestimated. For example, politicians first in Bavaria and later in Switzerland voted in favor of studies to explore homeopathics as a possible antibiotic alternative [13].

### Homeopathy and antibiotics

Antibiotic-resistant bacteria and germs are emerging as serious medical problems. For example, the WHO estimates that the probability of dying from resistant germs is 64% higher than from non-resistant germs [13]. To solve this problem, researchers are looking for new therapeutic approaches and antibiotic alternatives. As homeopathy was also recently brought increasingly and specifically into focus as an antibiotic alternative, this point will be discussed in more detail here. The fact that representatives of homeopathy are promoting it under the banner of the antibiotic resistance problem is not a new development, however. It should be mentioned here that the first discovery of a pathogen as the cause of a disease (*Bacillus anthracis*, anthrax, discovered by Robert Koch in 1876) occurred well after the postulation of homeopathy (1796). The work of Robert Koch, as well as that of Ignaz Semmelweis and Louis Pasteur, is now considered a scientific refutation of Hahnemannian homeopathy [16]. Currently, no valid scientific studies exist that justify homeopathy as an alternative to antibiotics.

When investigated by scientifically sound approaches, homeopathy does not have any effect beyond a placebo [2, 4, 17]. The placebo effect is irrelevant in serious bacterial infections. Studies that at first glance attribute a positive effect to homeopathy (currently around 4000 studies [10]) show clear deficits and bias on closer examination. Nevertheless, the unbroken social reputation of homeopathy is a worrying problem. The best example of this reputation is the aforementioned decision by the Bavarian parliament to commission a study to explore the potential of homeopathy as an alternative to antibiotics. This decision is not compatible with the overall negative evidence for homeopathy and consequently has been strongly criticized several times [13, 16].

## Add-on: homeopathy—The remedies and what is “inside”

Homeopathy recognizes potentially any source material as a candidate for a cure. Hahnemann did distinguish between the plant, animal and mineral kingdoms, but in doing so he only wanted to cover the totality of the forms of existence without prioritizing one or more of them.

As he focused on the spiritual medicinal power inherent in the substances and not on material interactions, it is not surprising that his exegetes in modern times even expanded this spectrum. They added things like electromagnetic radiation (e.g. several types of light) and the like to the possible source substances, mostly in the erroneous assumption that this was not a matter of material from the outset, but of (undefined) information or energy. These initial substances are generally referred to as imponderables. Substances, often artificially produced and/or mixtures, for which an association is made between their everyday use and their alleged suitability for curing certain symptoms are also usually referred to as imponderables. Examples are Berlinum moratium (Berlin Wall) for feelings of abandonment, separation and repression or gunpowder in cases of inflammation of skin and tissue wounds, even sepsis. Such purely associative symptoms, not found in a homeopathic remedy test, are not compatible with Hahnemann’s teaching.

In this respect, the spectrum of homeopathic source materials is potentially unlimited. As a result, several thousand monopreparations alone (which follow Hahnemann’s guideline to always use only one single remedy) are available. In Table 1, some ingredients of known globules are mentioned. It becomes clear that modern homeopathy has far more ingredients than simple medicinal plants [18].

**Table 1:** Table showing the breadth of possible ingredients of homeopathy. Examples are listed. The examples are sorted according to the different homeopathic kingdoms

**Plant kingdom**	
Acidum formicicum globules	Formic acid
Adonis vernalis globules	Adonis
Allium sativum globules	Garlic
Baptista globules	Wild indigo
Belladonna globules	*Atropa belladonna*
Bellis rerennis globules	Daisy
Candida albicans globules	Yeast
Digitalis globules	Red thimble
Eucalyptus globules	Blue eucalyptus
Nux vomica globules	Poison nut, Jatropha
Okoubaka globules	Bark of the West African jungle tree (*Okoubaka aubrevillei*)
Opium globules	Opium poppy
**Animal Kingdom**	
Apis mellifica globules	Honeybee
Bufo globules	Common toad
Falco pelegrinus globules	Peregrine falcon
Lac defloratum globules	Skimmed cow milk
Lachesis globules	Venom of the South American bushmaster snake
Latrodectus mactans globules	Black widow
Naja tripudians globules	Indian cobra
Pel myotis globules	Moleskin
**Mineral Kingdom**	
Calcium carbonicum globules	Oyster shell lime
Ferrum metallicum globules	Iron
Glonoinum globules	Sulfuric and nitric acid
Hamamelis globules	Virginia witch hazel
Jodum globules	Iodine
Kalium chloratum globules	Potassium chloride
Magnesium sulfuricum globules	Magnesium salt
Mercurius cyanatus globules	Mercury cyanide compound
**Imponderables**	
Electricitas globules	Electricity
Glyphosathe globules	Glyphosate herbicide
Luna globules	Lunar light (from different moon phases)
Magnetic poli ambo globules	Magnetic North Pole and Magnetic South Pole (already described by Hahnemann himself)
Positronium globules	Positively charged electrons
Sol globules	Sunlight
X‑Ray globules	X‑rays

Despite a great number of so-called complex remedies offered, combining two or more substances contradicts Hahnemann’s principle of the only and one single remedy (*unitas remedii*), often named as one of Hahnemann’s basic laws.

Special forms are so-called nosodes, which follow a special nomenclature that is not fully compatible with the basic homeopathic idea of the simile but is closer to isopathy (the cure with a “same*”*, Latin name *per idem*) [19]. Hahnemann rejected the idea of healing *per idem*, i.e. isopathy, in his final 6th edition of the Organon as “contrary to all common sense and therefore also to all experience”. Nevertheless, nosodes are widely accepted as part of the homeopathic universe.

- Nosodes of pathogenic origin: according to the original idea of the founder of homeopathy in America, Constantin Hering (1800–1880), are prepared from the pathogens or excretions of infectious diseases; these are used e.g. for the so-called homeopathic vaccination. The incompatibility with Hahnemann’s homeopathy here is also that the latter knows no prophylaxis but wants to intervene in an already detuned spiritual life force. This kind of nosodes is only offered in high potencies (C30, C200 and the higher LM potencies) [17]. Hering’s first nosode, Psorinum (from pus of scabies sores) is still in the repertoire of homeopathy today.
- Autonosodes: called *sarcodes* in homeopathy and also in the *Homeopathic Pharmacopoeia*, referring to their origin from body tissues. Autonosodes are prepared from bodily substances of the individual patient, perhaps best known are the placenta nosodes. Pathologically altered bodily substances, as in Hering’s nosodes, play no role here, with a few exceptions. Here we actually see isopathic remedies in their pure form. Their application shows a wide range, which is not limited to pathologies.
- Vaccine nosodes: vaccine nosodes are prepared from vaccines (not pathogens as with Hering’s nosodes), as classical homeopathic remedies according to the principle of similarity, which are used against complaints that may occur after vaccinations (drainage, detoxification), which is most compatible with classical homeopathy and also has nothing to do with prophylaxis; however, the question should be raised how the vaccine nosode knows what the complaints are and what the desired reactions of the immune system in the patient are [19, 20].

## Benefit or harm?

It is obvious that the widespread reputation of homeopathy as a gentle, side effect-free and above all effective medicine raises considerable problems given the established facts. For a long time, despite the knowledge of the medical irrelevance of homeopathy, scientific and medical circles did not focus on the issue because it was assumed that the use of homeopathy did not benefit but did not harm either.

This has become untenable today. Of course, as a specifically ineffective method based on scientifically untenable premises, homeopathy has the potential to mislead and harm patients. It is no longer justifiable to take an indifferent or neutral stance on the subject of homeopathy. Reasons of scientific, especially medical, ethics require that homeopathy propaganda be publicly and argumentatively resisted in the interest of patients.

The World Medical Association has taken a clear position in its *Declaration on Pseudoscience and Pseudotherapies in the Field of Health* in 2020 [21]:

Pseudoscience and pseudotherapies represent a complex system of theories, assumptions, assertions and methods erroneously regarded as scientific, they may cause some patients to perceive a cause-and-effect relationship between pseudotherapies and the perception of improvement, hence they may be very dangerous and are unethical. […]

Many countries lack the regulatory framework to address these pseudotherapies, which has allowed their proliferation. In the past, the medical profession considered them to be harmless due to their perceived lack of side effects, but nowadays there is enough evidence to suggest that they can pose a risk to patient safety. Pseudoscience and pseudotherapies may have significant potential risks and harms for various reasons:

- There is a risk that patients abandon effective medical treatment or prevention measures in favor of practices that have not demonstrated therapeutic value, sometimes leading to treatment failure for critical conditions that may even lead to death.
- There are frequent likelihoods of dangerous delays and loss of opportunity in the application of medicines, procedures and techniques recognized and endorsed by the scientific medical community as evidence-based effective interventions.
- They may cause patients to suffer financial damages, psychological and physical trauma, and go against the dignity of people, threatening their moral integrity.
- Unproven therapies may contribute to the rising of healthcare procedures.

This is absolutely unequivocal. All those who care about patient welfare should therefore take every opportunity to counter pseudo-medicine, especially the widespread and popular homeopathy, in medical practice, academic, research and public health systems with enlightenment and education.
